# Dynamics of Regulatory Networks in the Developing Mouse Retina

**DOI:** 10.1371/journal.pone.0046521

**Published:** 2012-10-03

**Authors:** Woochang Hwang, Laszlo Hackler, George Wu, Hongkai Ji, Donald J. Zack, Jiang Qian

**Affiliations:** 1 The Wilmer Eye Institute, Johns Hopkins University School of Medicine, Baltimore, Maryland, United States of America; 2 Department of Molecular Biology and Genetics, Johns Hopkins University School of Medicine, Baltimore, Maryland, United States of America; 3 Department of Neuroscience, Johns Hopkins University School of Medicine, Baltimore, Maryland, United States of America; 4 McKusick-Nathans Institute of Genetics, Johns Hopkins University School of Medicine, Baltimore, Maryland, United States of America; 5 Department of Biostatistics, The Johns Hopkins University Bloomberg School of Public Health, Baltimore, Maryland, United States of America; Universitat Rovira i Virgili, Spain

## Abstract

Understanding gene regulation is crucial to dissect the molecular basis of human development and disease. Previous studies on transcription regulatory networks often focused on their static properties. Here we used retinal development as a model system to investigate the dynamics of regulatory networks that are comprised of transcription factors, microRNAs and other protein-coding genes. We found that the active sub-networks are topologically different at early and late stages of retinal development. At early stages, the active sub-networks tend to be highly connected, while at late stages, the active sub-networks are more organized in modular structures. Interestingly, network motif usage at early and late stages is also distinct. For example, network motifs containing reciprocal feedback regulatory relationships between two regulators are overrepresented in early developmental stages. Additionally, our analysis of regulatory network dynamics revealed a natural turning point at which the regulatory network undergoes drastic topological changes. Taken together, this work demonstrates that adding a dynamic dimension to network analysis can provide new insights into retinal development, and we suggest the same approach would likely be useful for the analysis of other developing tissues.

## Introduction

Understanding gene regulation is essential to elucidate the molecular basis of human development and disease. Retinal development is an ideal model system that is tightly controlled through a variety of regulatory mechanisms such as transcriptional regulation, alternative splicing, and microRNA (miRNA) regulation. Dysregulation of any of these processes can lead to retinal disease [1], [2], [3]. To understand the molecular basis of retinal development and diseases, one traditional approach is to identify individual genes responsible for either retinal diseases or the developmental process. However, retinal cells must also deal with challenges such as how to maintain a phenotype in a stochastic and changing environment. Individual genes are not well suited to such challenges. Instead, several genes (or gene products) often form molecular circuits to carry out information processing functions, especially dynamic functions that change with time and place, as is common during development.

It has recently been found that transcriptional networks often contain recurring regulation patterns, termed *network motifs*. These network motifs can be considered the basic units, or recurring circuits, upon which the networks are built. These network motifs were computationally identified as patterns that occur more often than would be expected from a random distribution [4], [5], [6]. One example of a network motif is a feedback loop where two transcription factors (TFs) regulate each other. Understanding the functionality of these network motifs can help elucidate basic design principles and provide insight into the behavior of regulatory networks.

Different types of regulatory networks utilize different network motifs. For example, sensory regulatory networks make rapid and reversible decisions in response to environmental changes. It has been found that feed-forward loops, where one regulator regulates another regulator and both of them co-regulate a third gene, are extensively used in sensory regulatory networks. Experimental and theoretical analyses have suggested that feed-forward loops carry out interesting functions such as response acceleration, filtering of noisy input signals, and pulse generation [5], [7], [8], [9], [10], [11]. Distinct from sensory networks, developmental regulatory networks operate along a longer time horizon. They make decisions that are generally irreversible, and act on a time scale of several cell generations. In developmental networks, there is often an over-representation of feedback loop motifs [12], [13], [14]. Feedback loops can have two stable steady states: ON or OFF. Such a bi-stable switch can play an important role in the cell fate decision process during development by providing a lock-on mechanism [12], [13], [14].

Different types of gene regulation play critical roles in development and cellular homeostasis. One important class of regulators in gene regulatory networks is TFs. Previous studies have investigated the regulatory networks controlled by TFs (e.g., [15], [16]). Over the past several years, miRNAs have emerged as another important class of regulatory factors, and they are distinct from TFs in that they modulate gene expression at the posttranscriptional level [17], [18]. There is increasing evidence that these two classes of trans-acting factors, TFs and miRNAs, can work cooperatively [19], [20], [21]. The crosstalk between different types of regulators (e.g., TF and miRNAs) can be studied in the framework of network motifs. Several groups have provided new insights into their biology by identifying and characterizing interactions between miRNAs and TFs [22], [23], [24], [25]. For example, the transcription factor Yan represses the expression of a miRNA, miR-7, whereas miR-7 represses Yan expression. This reciprocal negative feedback loop facilitates exclusive cell fate determination during development, with Yan in progenitor cells and miR-7 in photoreceptor cells. Further studies suggested that miR-7 participates in several interlocking feedback and feed-forward loops to buffer against environmental variation during development [26].

In systems biology, understanding how biological molecules interact with each other to mediate biological function has become one of the challenging problems of the post genomic era. Many studies have been performed to understand biological systems based on observations of their topological metrics [27], [28], [29]. However, most of these analyses have been limited to static “snap shots” despite the reality that real world systems are generally both intrinsically and extrinsically dynamic. Adding the dynamic nature of biology to such analyses has the potential to add important new insights. For example, a genomic analysis of yeast regulatory network dynamics provided a new insight showing a large topological difference between endogenous and exogenous sub-networks [30].

To understand the underlying principles of regulatory networks containing transcriptional and post-transcriptional regulation, we analyzed the dynamics of regulatory networks in the developing mouse retina. Our study discovered diverse topological features and network motifs in the retinal regulatory network. Expression correlation between miRNAs and their predicted targets was not limited to negative correlations, suggesting complex underlying regulatory relationships. The active sub-networks of early and late time points were distinguished by their topological metrics and network motif usage. Emerging network motifs at particular developmental time points were found to carry out indispensable functions for that time point. Our study provides biological insights into the organization and reprogramming of the regulatory networks during retinal development.

## Results

### Gene Expression Profiling for Protein-coding Genes and miRNAs in the Developing Retina

**Figure 1:**
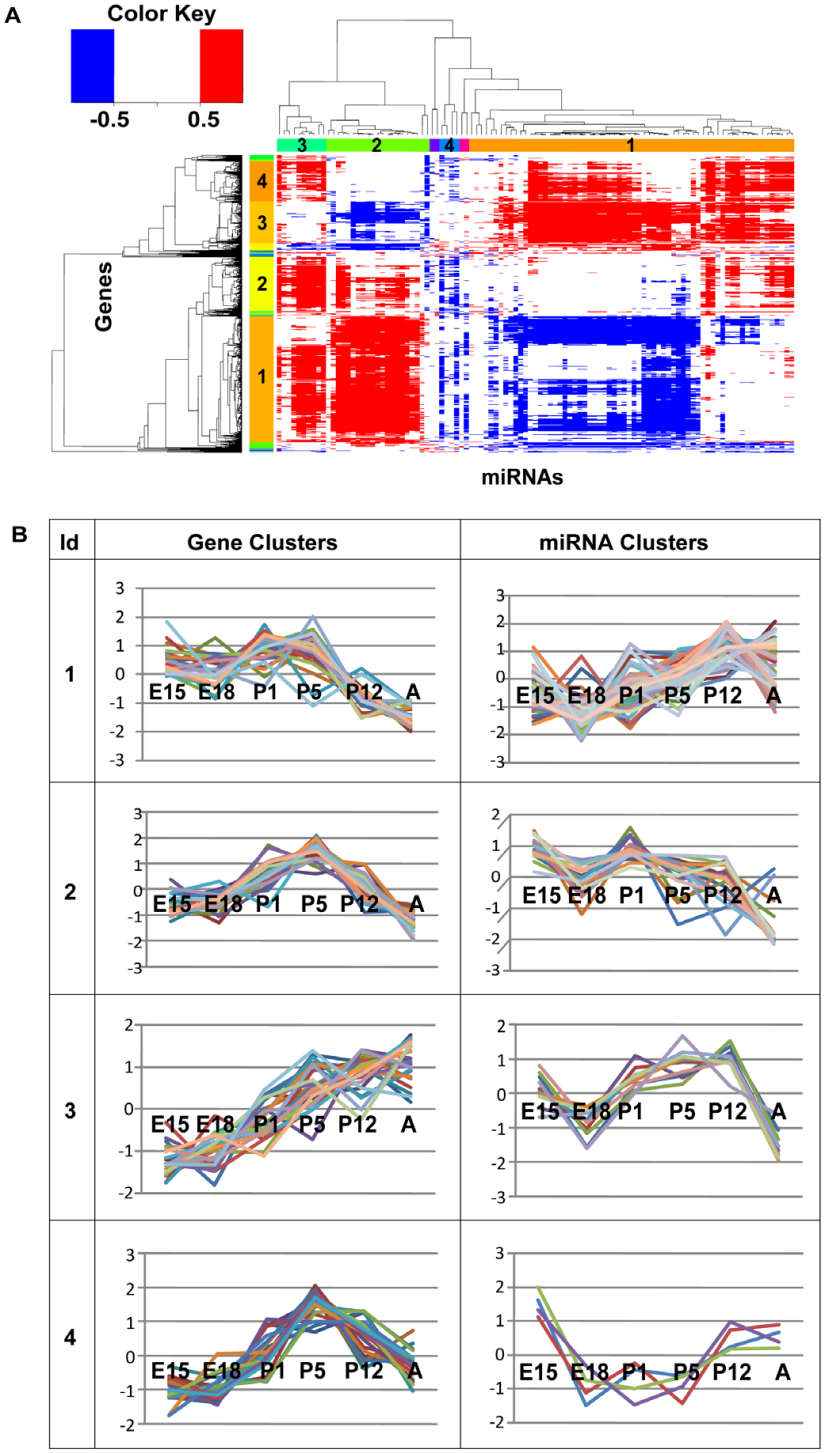
Expression correlations and clusters of miRNAs and genes. (A) Expression correlations between genes and miRNAs in the developing mouse retina. Rows represent protein-coding genes and columns represent miRNAs. Only the high correlations, bigger than 0.5(red) and less than −0.5(blue), are colored. The cluster IDs of gene and miRNA clusters are shown on each axis. (B) Gene and miRNA clusters. Relative expression of the 4 biggest clusters for genes and miRNAs throughout the developmental time points. Y-axis is the expression level in log2 scale. Each line in the clusters represents one gene.

For this study, we measured the gene expression of 356 miRNAs and 15,970 protein-coding genes simultaneously at 6 retinal developmental time points (embryonic day 15, embryonic day 18, postnatal day 1, postnatal day 5, postnatal day 12, and adult) [31], [32]. The expression profile provides the foundation for our analysis of regulatory network dynamics. We first analyzed the expression profiles of both protein-coding genes and miRNAs. Pearson correlation coefficients (cc) between miRNAs and genes were computed based on their expression profile over the six development time points and they were grouped according to their expression correlations (Figure 1A). Four gene clusters with more than or equal to 100 genes and 4 miRNA clusters with more than or equal to 4 miRNAs were identified. Each detected cluster showed distinct expression patterns peaking at different time points (Figure 1B).

**Figure 2:**
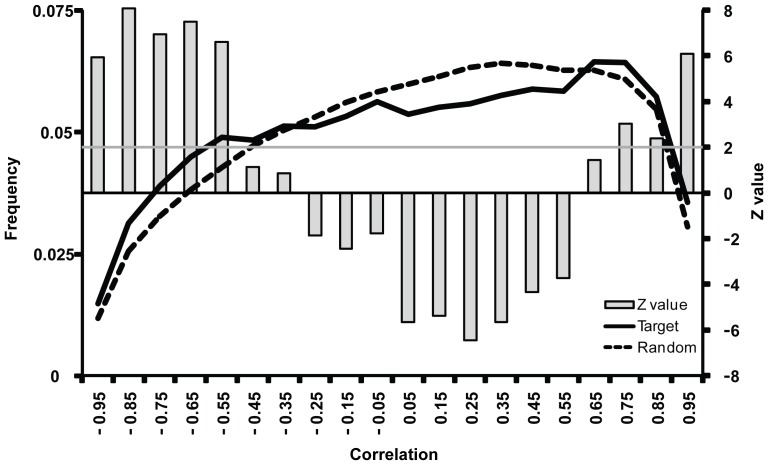
Expression correlations between miRNAs and their predicted targets. The distributions for the predicted targets in TargetScan database (solid) and for the random targets (dot) are shown. The column plot represents the Z-values of each interval against 1000 miRNA target randomization on the right Y-axis.

One obvious feature shown in Figure 1A is that protein-coding genes and miRNAs can have both positive (red) and negative correlation (blue). We then focused on the gene expression relationships between miRNAs and their predicted target genes based on TargetScan [33]. (The same target gene relationships will be used to construct the regulatory networks in the next section.) The distribution of the expression correlation between miRNAs and their predicted targets was compared and plotted in Figure 2 (the left Y axis). As control, we also calculated the correlation distribution between miRNAs and randomly selected genes with the same number of the predicted targets for a given miRNA. The randomizations were performed 1000 times and a Z-value was measured to show the statistical difference between the actual and expected correlation distribution (the right Y axis in Figure 2). The Z-value reflects the difference of the observed and expected occurrence of a correlation in the unit of the standard deviation from random simulation. As expected from the nature of miRNAs, highly negative correlation (cc<−0.5) of gene expression between miRNAs and predicted target pairs was enriched (Z>2.0), and the positive correlation (0.05<cc<0.55) was depleted (Z<−2.0). Surprisingly, the observed distribution was enriched (Z>2.0) at the highly positive range (cc>0.7) when compared to the random expectation.

Taken together, our analysis revealed that gene expression relationships between miRNAs and protein-coding genes, especially their target genes, are complex, including both correlated and anti-correlated relationships. Our finding suggested that these molecules might form interconnected networks in which different types of regulators influence all types of genes.

### Active Sub-networks During Retinal Development

To analyze the dynamic properties of the regulatory networks, a static integrated regulatory network (IRN) for mouse was built by combining several data sources, including ChIP-chip, ChIP-Seq, and miRNA target prediction databases. The regulatory target genes of TFs were predicted from ChIP-chip and ChIP-Seq experiments [34], while the target genes of miRNAs were acquired from TargetScan database [33]. The network includes 44 TFs, 284 miRNAs and 17,260 protein-coding genes, which form 139,987 TF-target and 36,024 miRNA-target relationships.

The active sub-networks at each time point were extracted from the static IRN using the gene expression data. The initial active gene set at a particular time point was defined as the set of genes and miRNAs showing expression that was higher than a specified threshold at that time point (see Methods). Then, the regulators, i.e. the TFs and miRNAs, of the genes and miRNAs in the active set were added to the active set regardless of their expression levels at the specified time point. This backtrack process was repeated until no more new regulators were added [30].

**Figure 3:**
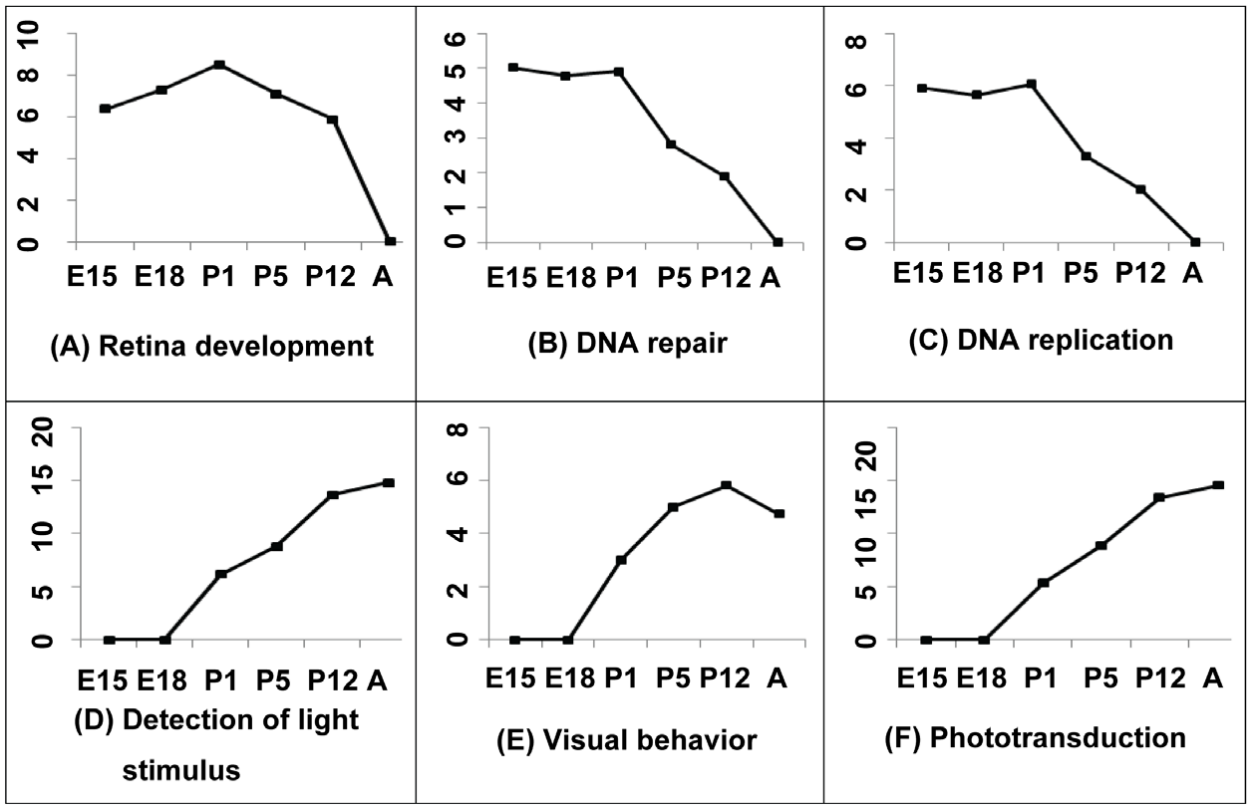
Enrichment analysis on six Gene Ontology terms (biological processes). Enrichment analysis for the genes specifically expressed in six development time points. X-axis is the time points and Y axis is the e-value of the terms at each time point.

To examine whether the active sub-networks are of biological relevance, we observed the functional features of the active sub-networks at each time point. After excluding the “housekeeping” genes, defined as genes that were expressed at all time points, we obtained genes that are specifically expressed at each time point. In total, 1234, 1295, 1294, 1553, 1336, and 1231 genes were specifically expressed at E15, E18, P1, P5, P12, and adult, respectively. Enrichment analysis on the Biological Processes from Gene Ontology (GO) was performed on these genes of all time points (Figure 3). An e-value (see details in Methods) was used to measure the enrichment of a gene set for a particular GO term. The genes specifically expressed at early time points were highly enriched for the retina development function (Figure 3A). Interestingly, genes expressed in early time points were also enriched for DNA replication and repair processes (Figures 3B and 3C). This finding correlates well with the highly proliferative state of retinal cells during early development, suggesting that active DNA replication and repair functions are particularly important for early developmental processes. Also making biological sense, the enrichment analysis of the functions related to light stimulus processing presented totally opposite behaviour, suggesting their importance at late stages in development (Figures 3D–3F). These functions were gradually enriched over time, and peaked on or after P12. This functional analysis of the sub-networks suggested that our definition of the active sub-networks was biologically relevant.

### Active Sub-networks for Early and Late Time Points are Structurally Distinct

We then analyzed structural and biological characteristics of the active sub-networks at the different time points. Our analysis demonstrated that the active sub-networks of early time points were clearly distinguished from those of late time points, both structurally and functionally.

**Figure 4:**
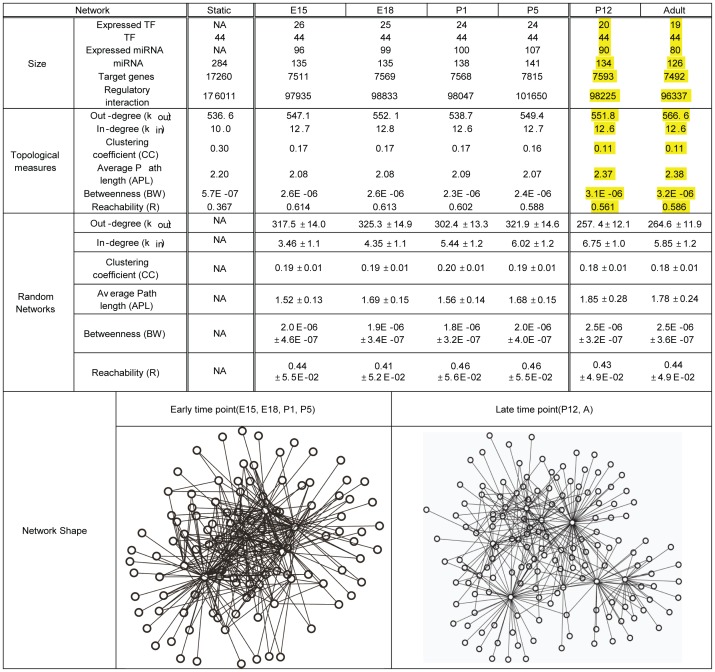
Topological measures of the static and active networks of six time points. Out-degree is the number of regulated genes by a TF or miRNA. In-degree is the number of regulating TFs or miRNAs of a target gene. Clustering coefficient measures the inter-connectivity around a node. Average path length is the average length of all shortest paths among all node pairs. Betweenness is the average number of shortest paths between all node pairs passing through a node. Reachability is the fraction of nodes that can be reached from a node in the network. The mean and standard deviation (mean±SD) of 300 random networks for each time point are presented in Random Networks row. Examples of early and late time point active sub-networks are illustrated in the last row.

The topological properties of the static IRN and the active sub-networks of each time point are summarized in Figure 4. The active sub-networks for early and late time points include similar number of TFs, miRNAs, target genes, and regulatory relationships. Furthermore, the average number of targets regulated by a TF or miRNA (out-degree) and the average number of TFs and miRNAs regulating a target (in-degree) of each time point are about the same. Thus, the active sub-networks for each time point were similar in size.

Despite the similar sizes of the active sub-networks, some topological measurements showed interesting differences between early and late time points (Figure 4). First, clustering coefficient (CC), which measures the extent of the interconnectivity among the directly connected nodes with a node of interest, is higher in the active sub-networks of early time points (E15, E18, P1 and P5) than those of late time points (P12 and adult). This observation suggests that (1) the active sub-networks at early time points are less organized in modular sub-structures and, (2) the IRNs underwent significant reprogramming between P5 and P12.

The higher CC leads to lower average path length (APL) and betweenness (BW) at the early time points. A node in an early time point active sub-network can be reached in smaller number of hops than in the late time point active sub-networks. The active sub-networks at early time points were decentralized showing lower BW because lower BW indicates that information in a network travels through diverse routes. In contrast, the late time point active sub-networks have higher BW, suggesting that information is controlled by a number of central nodes (hubs) in these networks. Lastly, better reachability (R) was observed in the early time point networks. In other words, more nodes can be reached from any node in the early time point active sub-networks. To provide an intuitive view of the difference between these two types of networks, we constructed two toy examples of sub-networks with similar properties of active sub-networks at early and late time points (shown in last row of Figure 4).

To ensure that these topological differences are significant, the same topological analysis was performed on a set of random networks with the same sizes of actual active sub-networks. The same number of TFs, miRNAs, and genes in the active sub-network for a time point were randomly selected from IRN and the same backtrack and wiring methods were used to construct a random sub-network for each time point. The topological metrics were averaged over 300 random sub-networks. The mean and standard deviation from 300 random networks are provided in the Figure 4. Most of the topological metrics except CC of the active sub-network in each time point were significantly different from those of the random networks. The TFs and miRNAs regulated more target genes and genes were regulated by more TFs or miRNAs in the active network than in the same size random networks. Higher CC in random networks indicates that the random networks were less modularized than the active sub-network at the same time point. In other words, the components in random networks were more inter-connected. The other metrics except CC indicated that the active sub-network at each time point was more centralized and modularized with better reachability than random networks. These measures demonstrate that the topological characteristics of the active sub-networks at each time point were significantly different from those of same size random networks, suggesting that the observed network structures are indeed biologically relevant.

To further understand the topological difference of the active sub-networks, we performed a network perturbation analysis. Robustness of a biological system against inner and outer stimuli is one of the important aspects from pathological interest. Isolated nodes (singletons) that were generated by sequential removal of nodes with highest degree (hubs) from sub-networks were numerated (Figure S1). More singletons were generated in the late time point sub-networks than the early time point sub-networks after more than 5 hubs were removed. This is because the late time point sub-networks have less connectivity between modules and a hub removal caused the module destruction and isolated its satellite components. In contrast, some of the satellite nodes of a removed hub were kept connected with other nodes in highly inter-connected early time point sub-networks. This feature made the active sub-networks at early time points more robust against perturbations than the sub-networks at late time points. Taken together, our analysis revealed that the active sub-networks at early and late stages of retinal development are topologically different. The active sub-networks at early development stages were more closely connected, more decentralized, and more robust.

### Network Motifs were Differentially Utilized in Early and Late Time Points

Network motifs are identified and used to understand the functional characteristic of a network. Network motif detection finds sub-graph patterns that emerge more often than would be expected in random networks. To understand the structural modularity and the functional dynamics of the retinal regulatory network, we performed network motif analysis for each active sub-network.

Network motifs of size 3 containing at least one TF and one miRNA were identified and classified into a corresponding motif. The statistical significance was calculated by comparing the occurrences of each motif in an active sub-network and random networks. An active sub-network is permuted to generate degree preserving random networks keeping the same incoming (i.e., number of upstream TFs and miRNAs regulating a node) and outgoing degree (i.e., number of downstream targets regulated by the node) with the same compositions of direct neighbors for each node in the network. The Z-value of a motif is calculated as the difference of its observed occurrence in an active sub-network and its averaged occurrence in several hundreds random networks, normalized with the standard deviation (see details in Methods).

**Figure 5:**
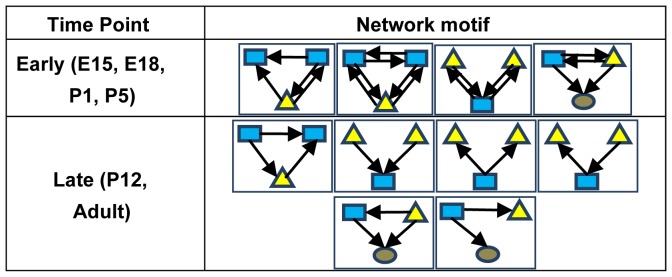
Overrepresented network motifs in early (E15, E18, P1, P5) and late (P12, Adult) time points. Blue rectangles are TFs. Yellow triangles are miRNAs. Grey circles are non-TF genes.

Overrepresented motifs in early and late time point active sub-networks demonstrated interesting and distinct behavior (Figure 5). Regulating or regulated mutual loops (RML), the motifs that contain two regulators (TFs or miRNAs) regulating each other, were overrepresented in early time points. RMLs were proposed to provide robustness for developmental processes by increasing the response sensitivity to activate target gene expression [35]. Since these RMLs all contain two-element mutual regulating motifs, we next examined whether the two-element motifs are the most basic motifs for early developmental stages. The same enrichment analysis was also performed for mutual regulating motifs containing only two regulators (i.e. TFs and miRNAs). Interestingly, 2-element mutual regulating motifs were not enriched in the active sub-networks at early time points (Figure S2), suggesting that the RMLs with three elements are the most basic regulatory units in this context. In contrast, for the active sub-networks at late stages, the motifs with one-way regulations, such as single input modules (SIM) in which one regulator controls two targets, co-regulating modules (CRM) in which two regulators control one target, and feed-forward loops (FFL), were overrepresented (Figure 5). FFLs were found to be capable of implementing rapid transient pulsing or delayed filtering dynamics [6]. SIMs were frequently found in systems of genes that function to form a protein assembly or a metabolic pathway [6]. In SIMs, one regulator coordinately regulates expression of a group of targets with a defined order [6]. CRMs were suggested to be involved in parallel, combinatorial, and compensatory regulations of a single target. These motifs might play an important role in maintaining homeostasis in the adult retina.

### A Turning Point During Retinal Development

**Figure 6:**
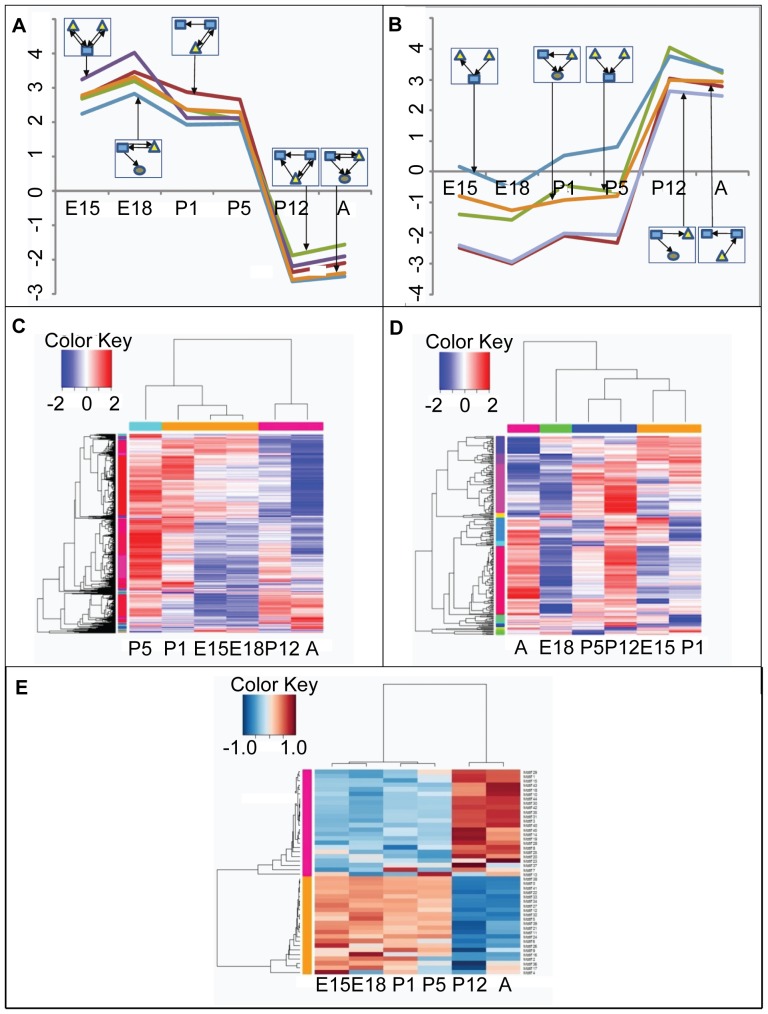
Network motif dynamics. Dynamics of two distinct patterns of network motif classes (A and B). Five network motifs are shown for each cluster. The network motifs are illustrated in the corresponding boxes. The expression of protein-coding genes (C), or miRNAs (D) cannot separate the early and late stages, while the Z-values for network motifs (E) show clear separation between early and late developmental stages.

An interesting phenomenon identified by our analysis is that there exists a turning point where the regulatory networks undergo significant reprogramming. As we observed with the differential topological properties of the active sub-networks between early and late time points, we found that the topological properties change between P5 and P12. A similar behavior for the Z-value dynamics for network motifs was also observed. Five representative motifs are illustrated in Figures 6A and 6B. Most of RMLs were overrepresented in early time points but underrepresented in late time points, while FFLs, SIMs, and CRMs behaved in the exact opposite direction. The Z-values of these motifs showed a steep difference between P5 and P12, suggesting that a reprogramming occurs during this period.

We then examined whether the reprogramming is inherently encoded in the gene expression profile during development. Figures 6C and 6D display the relative expression level of protein-coding genes and miRNAs throughout the developing period, respectively. The dendrograms in Figures 6C and 6D show that classifying developmental stages based only on gene or miRNA expression profiles is unable to identify the turning point. In contrast, the occurrences of network motifs (i.e. Z values) did show us the clear boundaries between early and late developmental stages (Figure 6E). In summary, our analysis showed that the turning point is the result of the molecular interactions in the regulatory networks rather than an automatic consequence of gene expression changes during the development.

## Discussion

Expression profiles of miRNAs and genes for six time points in the developing mouse retina were analyzed. Genes and miRNAs were grouped according to their expression pattern throughout the six developmental time points. The identified gene clusters presented distinguished expression patterns peaking at different time points. Identified gene clusters and their enriched biological processes make biological sense. For example, a gene cluster with expression peaking at early developmental time points was enriched in developmental functions. In addition to this, miRNAs presented interesting expression correlations with genes in the dataset. For example, the cluster containing genes that were maximally expressed at early time points (E15, E18 P1, P5) was negatively correlated with the miRNA cluster that showed increased expression at late time points (P12, A). This pattern was consistent with the reasonable hypothesis that miRNAs that are expressed at later time points might suppress the genes associated with developmental functions. Conversely, the genes that are enriched in sensory and stimuli response-related functions, which peak at the late time points, might be suppressed by the miRNAs that are expressed at early time points. miRNAs are presumed to contribute in conferring proper functions by suppressing unwanted genes at appropriate time points. Our observation of complex relationships between mRNA and miRNA gene expression suggested that the regulatory relationships between these two types of regulators are inherently interconnected.

Active sub-networks for each developmental time point were built and analyzed to study the interacting dynamics, i.e., network motifs, among TFs, miRNAs and target genes in the developing mouse retina. To define the active sub-networks, we used a backtrack algorithm. We first determined an initial gene set that is highly expressed. The backtrack algorithm then identified additional genes that are upstream regulators of the genes in the initial set. These additional genes are not highly expressed, but are considered to be active. The reason is that some active transcription factors are not necessary to be highly expressed. For example, CRX, a well-studied transcription factor in retina that regulates the expression of rhodopsin, is not highly expressed. In addition, PAX6, a “master control” gene for development of eye, is also low expressed. If we only used the initial active gene set, these regulators known to be important for eye development will be excluded in our analysis. Therefore, we believe backtrack algorithm will help to define a comprehensive set of active genes at developmental stages.

Distinct network motifs were overrepresented at early vs. late time points. Motifs with RML were overrepresented at the early time points. In the late time points, SIMs, CRMs, and FFLs were overrepresented. We suggest that this differential use of motifs across development reflects the evolutionary selection of motifs whose network properties best fit the requirements of early versus late developmental processes. For example, robustness is secured by RMLs for developmental processes in the early time points and FFLs provide rapid response or delayed filtering dynamics against stimuli in the late time points. Furthermore, emerging patterns of network motifs provided a turning point between early and late time points in the developmental process. It was impossible to discover turning points between different stages by studying gene or miRNA expressions only. Our study suggested that network motif dynamics analyses among TFs, miRNAs and genes offer better understanding of the regulatory system in the developing mouse retina.

One future direction is to study the regulatory networks in distinct retinal cell types. Over a dozen different cell types are found in the retina, yet our analyses lumped them all together because the expression measurements we utilized were derived from total retina. Given the complexity and interconnectivity of distinct retinal cells, it seems likely that applying the methods described in this paper to data sets derived from individual cell types, such as rods, cones, bipolar, and retinal ganglion cells, would yield interesting regulatory networks with greater precision and resolution. Such studies might also provide insights about possible gene expression network interactions between neighboring cells, and how such interactions might change during development.

## Materials and Methods

### Regulatory Network Construction

The static integrated regulatory network (IRN) for a mouse was constructed by combining target prediction datasets for TFs and miRNAs. The static IRN includes 44 TFs, 284 miRNAs, 17,260 target genes, and 176,011 regulatory relationships among them. Gene and miRNA expression for the developing mouse retina was collected for six time points (embryonic day 15, embryonic day 18, postnatal day 1, postnatal day 5, postnatal day 12, and adult) [32]. Active sub-networks for each time point were extracted from the static IRN by incorporating these time series expression data. For each time point, genes, TFs, and miRNAs showing higher expression than a threshold were identified as the active set. All TFs and miRNAs regulating any gene in the active set were also included in the active set. The active sub-network of a time point was formed by wiring the active components if they had a regulatory relationship in the IRN.

### Functional Enrichment

The extent to which a cluster is associated with a specific biological function is evaluated using p-value and e-value. The p-value is the probability that a cluster would be enriched with genes in a particular function by chance alone.
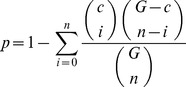
(1)
*c* is the size of the cluster containing *k* genes with a given function; *G* is the size of the universal set of genes and contains *n* genes with the function. All p-values were corrected for multiple hypotheses testing using Bonferroni method. An e-value is the ratio of the number of genes enriched with a function to the number of genes expected to be enriched with the function in a cluster.



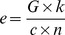
(2)The enrichment significance of a cluster with a function is decided based on p-value and e-value.

### Topological Metrics

Degree of node *v*, *k(v)*, in a network is the number of arcs incident to node *v*. The number of incoming arcs to a node and the number of outgoing arcs from a node are called in-degree and out-degree of the node respectively. Clustering coefficient of node *v* measures the extent of the interconnectivity among the directly connected nodes with the node. It is the ratio of the number of arcs among the direct neighborhoods to the number of arcs that could possibly exist among them.
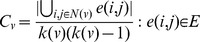
(3)
*N(v)* is the set of directly connected nodes with node *v*. *e(i,j)* is an arc from node *i* to node *j*. *E* is the set of arcs in the network. The path length between node *i* and *j* is defined as the number of arcs on the shortest path between them. Betweenness of node *v, BW(v)*, is the number of the shortest paths passing through node *v* out of the shortest paths from all nodes to all others.
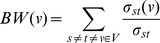
(4)σst is the number of the shortest paths between node s and t and σst(v) is the number of the shortest paths passing through node v out of σst. V is the set of nodes in the network. Reachability R of node v is the portion of other nodes that can be reached from the node. For all these metrics, the mean value of all nodes in a network was used to get the global view of the network.

### Network Motif Identification and Significance Analysis

All size-3 sub-graphs in a network are enumerated based on the algorithm developed by Wernicke [36]. An identified sub-graph is classified into a network motif if each corresponding node pair between a sub-graph and a motif has the identical type, i.e., TF, non-TF gene or miRNA, and the same number of incoming and outgoing arcs with the same compositions of incoming (number of TFs or miRNAs regulate the node) and outgoing (number of TFs, non-TF genes, or miRNAs regulated by the node) arcs. To evaluate the statistical significance, the occurrence of sub-graphs for each motif in real networks and random networks were compared. Degree-preserving random networks are generated as follows to evaluate the statistical significance. A real network is permuted to generate degree preserving random networks keeping the same incoming and outgoing degree with the same compositions of direct neighbors (i.e., number of TFs or miRNAs regulate the node and number of TFs, non-TF genes, or miRNAs regulated by the node) for each node in the network. For example, a random arc with the same type of connected nodes (from a TF t_2_ to non-TF gene g_2_) is chosen for a given arc (from a TF t_1_ to non-TF gene g_1_). These two arcs are swapped, i.e., connect t_1_ to g_2_, connect t_2_ to g_1_, and remove the arcs from t_1_ to g_1_ and from t_2_ to g_2_, if arcs from t_1_ to g_2_ or from t_2_ to g_1_ do not already exist. This arc permutation is repeated until a permuted network becomes random enough. The Z-value of a motif is calculated as the difference of its observed occurrence in a real network and its averaged occurrence in several hundreds random networks, normalized with the standard deviation.

## Supporting Information

Figure S1 **Network perturbation analysis.** Number of singletons that are generated in function of sequential removal of highest degree nodes from the active sub-networks.
(TIF)

Figure S2 **2-element mutual regulating motif enrichment analysis.** Z-values for 2-element mutual regulating motif compared with 1000 degree preserving random networks in the sub-network at each time point.
(TIF)
