# Supplementary figures and images for: Dynamics of Regulatory Networks in the Developing Mouse Retina
Source: PLoS One. 2012 Oct 3;7(10):e46521. doi: 10.1371/journal.pone.0046521 (PMC3463606; doi:10.1371/journal.pone.0046521)

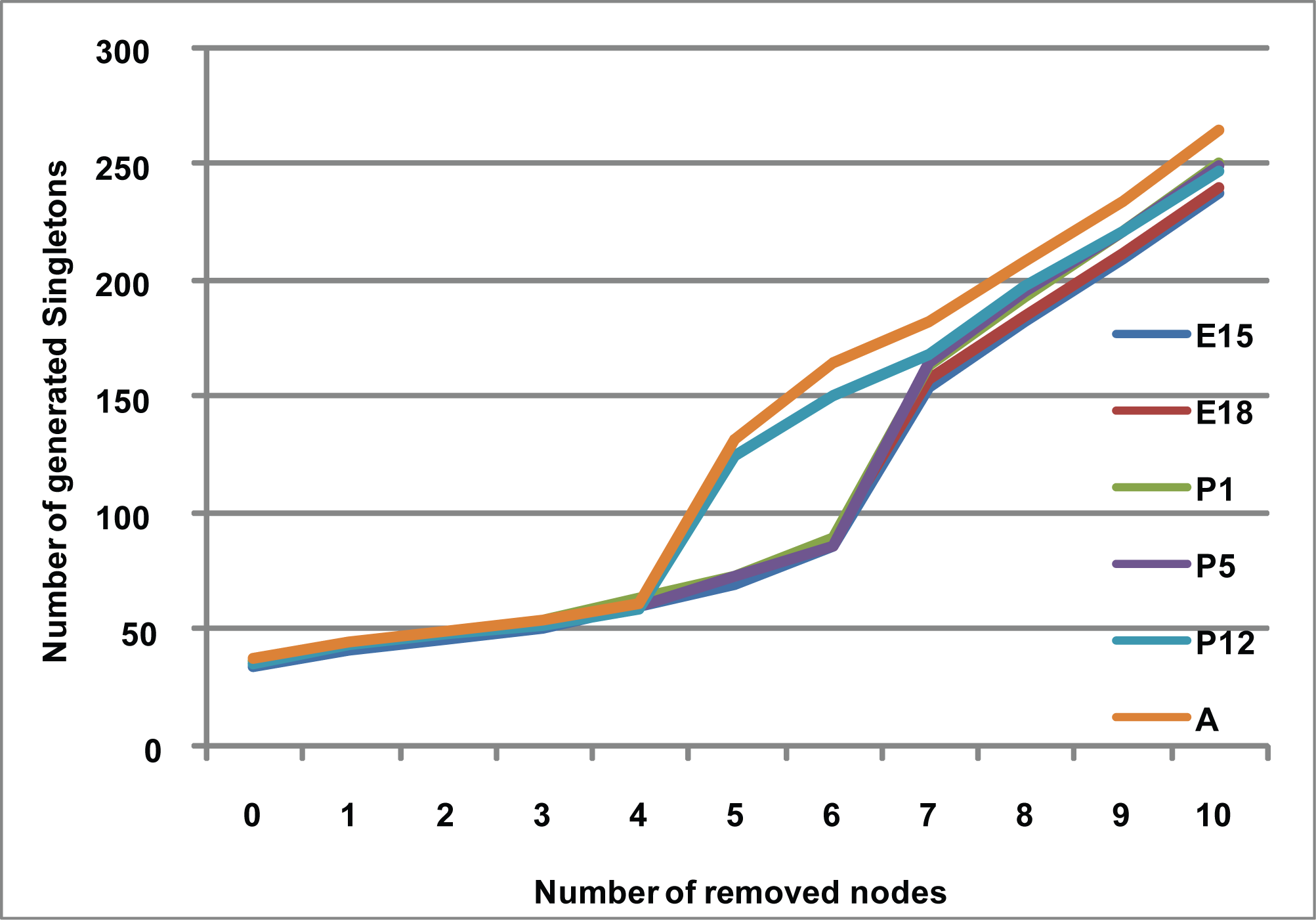

Supplement: Figure S1 — Network perturbation analysis. Number of singletons that are generated in function of sequential removal of highest degree nodes from the active sub-networks. (TIF) [file pone.0046521.s001.tif]

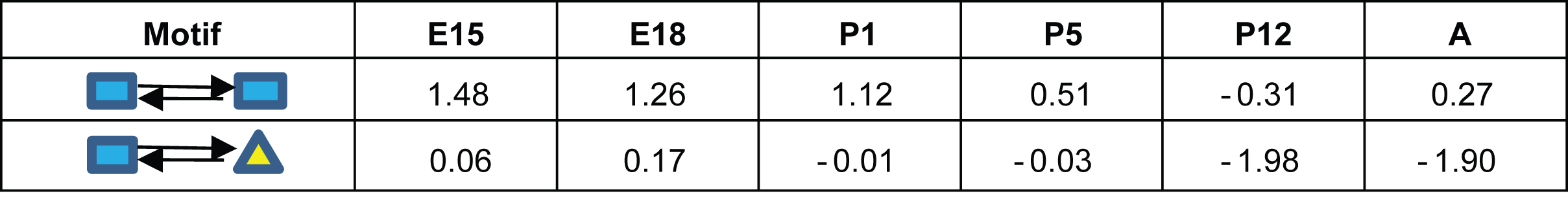

Supplement: Figure S2 — 2-element mutual regulating motif enrichment analysis. Z-values for 2-element mutual regulating motif compared with 1000 degree preserving random networks in the sub-network at each time point. (TIF) [file pone.0046521.s002.tif]
